# Parents' Views of Family-Centered Care at a Pediatric Intensive Care Unit—A Qualitative Study

**DOI:** 10.3389/fped.2021.725040

**Published:** 2021-08-25

**Authors:** Karina Terp, Janne Weis, Pia Lundqvist

**Affiliations:** ^1^Department of Health Sciences, Lund University, Lund, Sweden; ^2^Department of Neonatology, Copenhagen University Hospital, Copenhagen, Denmark

**Keywords:** family-centered care, pediatric intensive care, parent satisfaction, partnership, quality of care, qualitative approach

## Abstract

**Purpose:** To describe parents' views of family-centered care at a pediatric intensive care unit.

**Design and Methods:** A qualitative descriptive study with a deductive and inductive approach was conducted based on the principles of family-centered care. Inclusion criteria were parents of children cared for at a pediatric intensive care unit for at least 48 h. Parents of children who died during the hospital stay were excluded. The sample consisted of spontaneous responses from 70 parents to five open questions in the EMpowerment of PArents in THe Intensive Care questionnaire, which was completed at discharge. The spontaneous responses were analyzed using thematic analysis.

**Results:** The analysis of the parents' statement illuminated that partnership, the essence of family-centered care, appeared incomplete. Partnership was particularly evident regarding parents' experiences of being treated with empathy and respect. It also seemed prominent in situations where the professional team provided support to the child, parents, and family. Based on the parents' statements there was potential for development of the family-centered care approach in aspects such as decision-making concerning care and treatment, as well as improving person-centered communication on order to capture parents' experiences and needs in the highly technological pediatric intensive care unit environment.

**Conclusions:** Although in general parents were satisfied with the care, areas for improvement were identified such as participation in decision-making about care and treatment as well as person-centered communication. The results can contribute to future quality improvement interventions focusing family centered care at pediatric intensive care units.

## Introduction

Medical and technical development as well as improved nursing care have resulted in better outcomes and increased survival rates for children in need of pediatric intensive care. In Sweden, pediatric intensive care unit (PICU) admissions increased by 14% between 2010 and 2019 (1). One reason for the increased admission rates might be that improvements in pediatric care have resulted in more advanced medical care at a PICU. Another reason can be our growing population, as the child (0–18 years) population in Sweden increased by about 10% between 2010 and 2019 (2). Consequently, more parents and families are living through the pediatric intensive care experience.

Previous studies have shown that parents of children cared for at a PICU experience stress due to the unfamiliar environment with an intense atmosphere, technological medical equipment, and monitors with sounds and alarms (3, 4). Furthermore, the child's changed appearance and behavior due to her/his condition and medical treatment might negatively affect the parents (5, 6), placing them at risk of developing both acute- and post-traumatic stress disorder. Symptoms such as anxiety, difficulties concentrating and sleep problems have been reported (6–8).

Organizing pediatric intensive care in accordance with the principles of family-centered care (FCC) seems to be beneficial for parents both during hospitalization and post-discharge, as it supports the parental role and strengthens family functioning (9, 10). Coyne (11) describes that parents considered FCC important for their child's well-being as they found that their specific knowledge about the child was fundamental for the quality of the care provided (11). The Institute for Patient and Family-Centered Care emphasizes that the purpose of FCC is to achieve “partnership” between patient, families, and healthcare professionals (HCPs) characterized by mutual respect and dignity, sharing of professional as well as person-specific information, and inclusion of patient and family in the care as well as in decisions about care and treatment (12). FCC advocates parents' active participation in care planning and treatment which increases their confidence as well as satisfaction with care (13). Furthermore, parents, as well as nurses, reported that the child becomes more confident and calmer when a parent is nearby during the hospital stay (11).

Communication is essential in FCC as it affects the way information is shared between the HCPs, the parents, and the child. In some cases, the child is unable to speak for her/himself due to young age, sedative medicine, or care on a ventilator (14, 15). Foster et al. (16) showed that parents viewed good communication with HCPs as significant and wanted to be well-informed about their child's care during her/his hospital stay. In order to work in accordance with the principles of FCC, nurses need to change their professional approach from being the sole “expert” to inviting next of kin and the patient to participate in the planning and implementation of care (16). This highlights a need for knowledge and experience of communication styles, such as person-centered communication, which is based on a dialogue between the person being cared for, her/his next of kin, and the HCPs (17, 18).

It has also been established that parents' satisfaction with care increases when the healthcare team works according to the principles of FCC. Previous studies have shown that working in accordance with FCC improves both the child's and the parents' well-being, and increases the safety of the child (13, 19). Despite this knowledge, it appears to be difficult to fully implement FCC in practice (20). However, little is known about parents' views of FCC related to pediatric intensive care in Swedish settings. Parents' experiences can guide the further development, implementation and adaptation of FCC in the PICU context to meet both children's and parents' needs, leading to a sustainable outcome.

### Aim

To describe parents' views of family-centered care in a pediatric intensive care unit.

## Methods

### Design

In the present study, a qualitative descriptive method with a deductive and inductive approach (21) was used. The study was conducted as part of a larger study to psychometrically evaluate the EMpowerment of PArents in THe Intensive Care (EMPATHIC-30) questionnaire by Latour et al. (22) in a Swedish setting.

### Setting

The study was conducted in two out of the four Swedish PICUs. Each unit has about 350 admissions per year. Each of the study units contains 8–12 beds, where the variation in the number of beds is mainly due to organizational reasons such as reduced staffing during weekends and holiday periods. The units primarily treat children from within their own catchment area, although children in need of high technological care can be referred from hospitals outside the PICUs' catchment area. The units have unlimited access for parents, siblings, and other relatives. In the intensive care room, the parents cannot spend the night in a bed next to the child due to limited space but are offered a chair. However, all parents and siblings are offered accommodation at a Ronald Mc Donald house nearby. The units have both single and multi-bedded rooms. Parents are normally not present during the daily rounds but are informed by the physicians afterwards. They are usually invited to attend care procedures, including cardiopulmonary resuscitation. The physician responsible for the child may vary on a weekly basis due to the physicians' schedule. The healthcare professional team in this study comprises physicians, nurses, and assistant nurses.

### Sample

**Figure 1:**
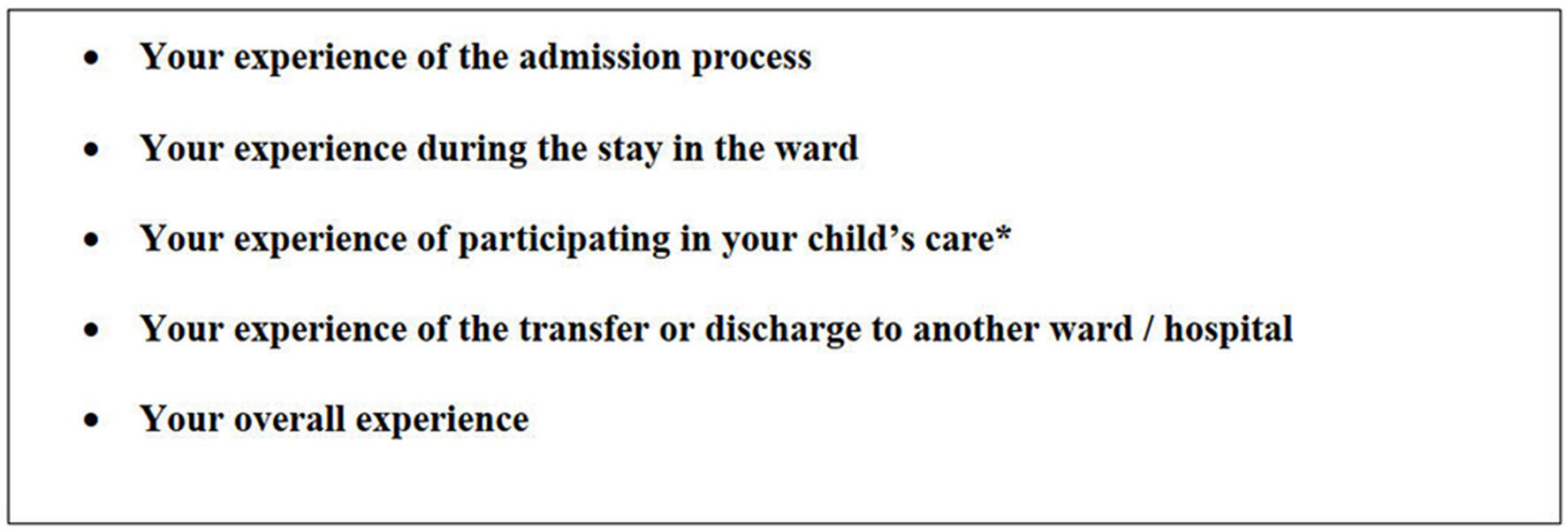
The 5 open-ended questions in the EMPATHIC-30. *Added questions in the Swedish version.

The sample consists of parents' spontaneous responses to the five open questions (Figure 1) in the Swedish version of the Empathic-30 questionnaire. The translation of the Swedish EMPATHIC-30 followed methodological procedures and psychometric evaluation. Inclusion criteria were parents whose child had been cared for at the PICU for at least 48 h and who had a good command of both spoken and written Swedish. An exclusion criterion was parents of children who died during hospitalization.

In total 234 questionnaires were distributed, and the response rate was 42.7% (*n* = 100). Three questionnaires were excluded due to not meeting the inclusion criteria (*n* = 2) or being returned blank (*n* = 1). Of the final 97 questionnaires, 72 % (*n* = 70) were included in this study (mothers: *n* = 40; fathers: *n* = 30). The 27 excluded questionnaires had either no responses to the open questions or they were just answered briefly with, e.g., good.

### Instrument

EMPATHIC-30 is an instrument measuring parents' satisfaction when their child is cared for at a PICU. The questionnaire consists of five domains that correspond with the concepts of FCC: information, care and cure, organization, parental participation, and professional attitude. The parents rate their experiences on a 6-point Likert scale ranging from 1 “certainly no” to 6 “certainly yes” or Not Applicable (NA). The original EMPATHIC-30 concludes with four open-ended questions inviting parents to describe their experiences of their time at the PICU in free text. In the Swedish version, one more question was added with a focus on the parents' experiences of participating in the child's care (Figure 1). The EMPATHIC-30 was developed and psychometrically evaluated in the Dutch language and showed a satisfactory result, with Cronbach's Alpha varying between 0.73 and 0.83 for the domains and 0.93 for the total scale (22). The instrument has been translated into other languages with acceptable psychometric properties (23, 24). Permission to translate and psychometrically evaluate the Empathic-30 was obtained from the original author (Latour J.).

### Data Collection

Parents who met the inclusion criteria received brief oral information about the study from the nursing staff at the ward in connection with the child's discharge from the PICU. Those who were interested in participating received an envelope with written information about the study, a document for informed consent, the EMPATHIC-30 questionnaire and a pre-stamped envelope. If parents had not been asked to participate before the child was discharged, a short letter informing them about the study and an envelope with information as described above were sent to them by post by a secretary at the PICU. Data collection took place from February 2018 to September 2020.

### Framework

The theoretical FCC framework in the present study was the “Patient- and Family-Centered Care” (PFCC) model as defined by the Institute for patient and family-centered care (12). According to the chosen framework, it is the patient and her/his family who define their family. The patient and family are viewed as essential allies in a mutually respectful partnership and decide the extent to which they wish to participate in care and decision making. The four core concepts in the framework are: Dignity and Respect, highlighting the importance of the professional team listening to the wishes of the patient and family, as well as respecting their values and cultural background; Information Sharing, which refers to collaboration through mutual information sharing between the patient, family, and professionals. The importance of continuous, honest, and accurate information is emphasized as necessary for integrating patients and families in decision-making; Participation, representing professionals' encouragement and support to motivate the patient and family to participate in care and decisions according to their own ability and desires; Collaboration includes the role of patients, families, the professional healthcare team, and healthcare leaders in policy and program development, research activities, and care delivery. Partnership is achieved when cooperation between patient, family, and HCPs is practiced in accordance with the core concepts in the framework (12).

### Data Analysis

First, a deductive thematic analysis guided by the core concepts of FCC in the PFCC model (12) was conducted in accordance with Braun and Clarke (21). Initially, familiarization with the data was obtained by reading and rereading the text. The data were then carefully reviewed and compiled in a separate document, providing an overview of the entirety of the data. The data were then deductively coded based on the PFCC core concepts: Dignity and respect, Information sharing, Participation, and Collaboration (12), which constituted the themes. Subsequently, the first author (KT) inductively sorted and coded the statements within each theme, and then grouped codes with a similar content into subthemes. All authors discussed the codes and subthemes until consensus was achieved (21).

## Ethical Considerations

The study was approved by the regional ethical review board in Lund (Ref 2018/547, 2019-04602) and was conducted in accordance with the Helsinki Declaration of 2013 (25). The parents recruited at the units received oral and written information before their child's discharge, while those who were included after their child's discharge received extensive written information. The written information stated that participation was voluntary, that they could withdraw at any time without giving a reason, and that confidentiality was ensured. Written informed consent was obtained.

## Results

A variety of patterns within the data emerged during the analysis of the 70 parents' statements pertaining to the open questions in the EMPATHIC-30. Parent characteristics are presented in Table 1. The children (*n* = 46) varied in age from newborn to 15-years and duration of their hospital stay varied between 2 and 84 days. The children's characteristics can be found in Table 1.

**Table 1:** Characteristics of participants included in the study.

	**Participants**	**Percentage**
	**(*n* = 70)**	**(%)**
**Parents**		
Mothers	40	58
Fathers	30	42
**Parents' age**		
Mean	37	
Range	32 (23–55)	
**Education level**		
• Primary school	2	3
• High school	24	34
• University/college	41	59
• Other education	2	3
**Ethnicity**		
• Swedish	63	90
• European	6	9
• Other	1	1
**Parents' previous experience of PICU**		
Yes	14	20
No	56	80
**Child's age** ***n*** **=** **47**		
• 0–6 years	43	91
• 7–18 years	4	9
**Length of stay**		
• 2 days	7	10
• 3–7 days	32	46
• 8–10 days	5	7
• >10 days	26	37
**Mechanical ventilation (** ***n*** **=** **47)**		
• Yes	44	93
• No	3	7

**Figure 2:**
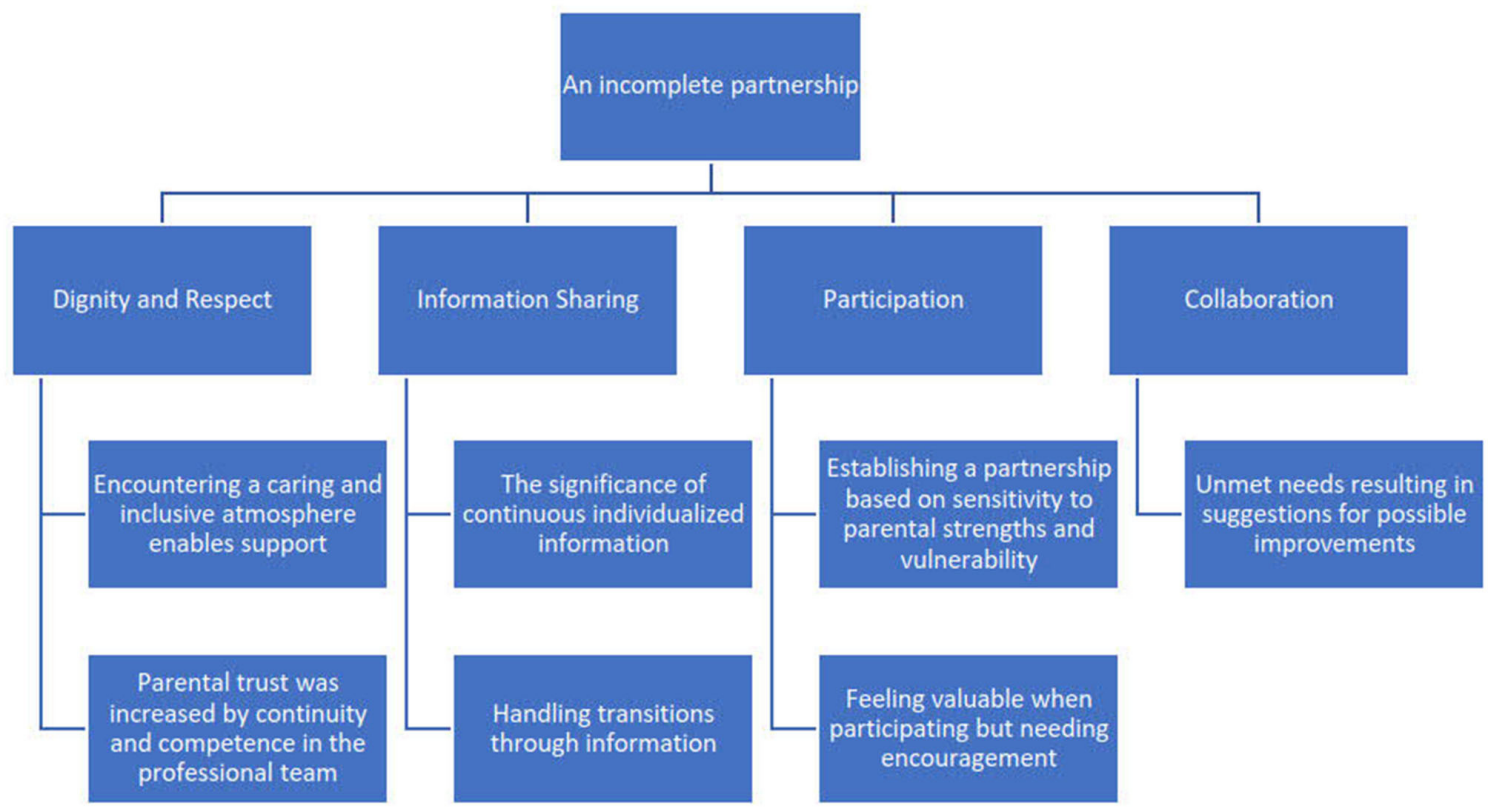
Overarching theme, main themes, and subthemes.

An incomplete partnership was the overarching theme reflecting parents' views of FCC at a PICU. An incomplete partnership emerged due to the fact that the content of parents' statements reflecting partnership appeared more prominent in some of the main themes than in others. Partnership became particularly evident in the parents' experiences of being treated with empathy and respect, as well as in situations where the professional team provided support for the child, parents, and family as a whole. However, there was potential for development when it came to achieving partnership in aspects such as taking an active role in decisions concerning care and treatment, as well as in person-centered communication that could capture parents' experiences and needs in the PICU's high technological environment. The overarching theme, themes, and subthemes are presented in Figure 2.

### Dignity and Respect

#### Encountering a Caring and Inclusive Atmosphere Enables Support

Although parents described a chaotic time and being in a state of shock, which made it difficult for them to handle the situation of having a child in need of pediatric intensive care, they felt well-cared for from their first encounter with the HCPs at the PICU. They experienced that the members of the professional team did their utmost for the child and family, even when they had a heavy workload. The parents felt supported in their overwhelming situation when they received attention from the professionals on the team, which in turn strengthened their relationship.

“*Despite all the difficult information, operations and such it felt wonderful to be surrounded by all those fine people.” (J)*

Although the parents considered the professional team treated them well and with respect, some felt that they needed more support and understanding. They described shortcomings, especially when the child had disabilities.

“*I think that it should be possible to adapt the care to the patient.” (G)*

The parents described feeling ignored when their expertise as a parent was not requested, such as how to best approach their child in caring situations. Parents also wished that some of the professionals would have exhibited a gentler approach toward their child.

#### Parental Trust Was Increased by Continuity and Competence in the Professional Team

Even though the parents experienced fear and anxiety about their child's condition, as well as the ongoing medical care and treatment, they found that the PICU itself created a sense of security. They described that the professional team delivered high-quality care. The professional team's competence induced confidence in the parents, even when emergencies occurred. They described the professional team as calm and acting in a way that created a sense of trust. When necessary, the parents felt safe about handing over their child's care to the professional team. They also felt able to leave the PICU in the knowledge that their child was well-taken care of.

“*I felt incredibly secure knowing that our child was receiving the best possible care.” (C)*

On the other hand, when contradictory opinions about the child's care and treatment occurred among the professional team, the parents became unsure about the professionals' competence, which was a source of stress. Frequent staff changes in the professional team around the child also created a sense of insecurity among parents. From their perspective, everything functioned best when the professional team members knew them and their child.

“*In my view one negative aspect is new staff all the time. Doctors as well as nurses. So much extra work for everybody.” (I)*

Discharge was associated with conflicting emotions. The parents found it difficult to leave the safe PICU environment and the highly competent professional team. Despite the fact that it was positive that the child had recovered and could be transferred to an ordinary pediatric unit, they felt anxious about having to take responsibility for the child's continued daily care. The need to create relationships with new staff members was also stressful for them.

“*Leaving the intensive care unit and coming to an ordinary ward always feels a little unsafe.” (U)*

Parents of children with disabilities experienced the high competence of the PICU staff as relief from their role as a main caregiver. They felt safe handing over the responsibility for their child's daily care to the professional team.

“*That was the first time I felt safe handing over responsibility” (K)*

### Information Sharing

#### The Significance of Continuous Individualized Information

The parents felt supported when they received continuous information from the professional team about their child's care. They felt they could ask questions without feeling foolish. If the child's condition changed, the professional team took the time to explain the current situation and go through the changes in care and treatment. The parents experienced that the professional team focused on the whole family, and when necessary, the information was even comprehensible for siblings.

“*…the staff members took the time to explain everything they did, even to the older sister.” (B)*

Parents also valued information about situations that occurred when they were not present, such as during surgery or transport between different units. In acute situations, the professional team members focused on the critically ill child and put dissemination of information on hold. However, as soon as the situation allowed, the parents reported receiving attention from a team member who informed them about the critical situation.

“*…when everything had calmed down and was over the staff members were very caring towards us and gave us a lot of information and support.” (A)*

Conversely, parents also mentioned times when they had not received adequate information or experienced that information had been withheld. They speculated that this might have been because the healthcare team assumed they could not comprehend the information. The parents expressed that no matter how bad the situation was they wanted the HCPs to share all information about the child's condition with them.

“*But we didn*'*t understand the seriousness of our child*'*s situation until it became VERY serious. If this was down to poor information or that we didn*'*t want to assimilate it, I don*'*t know. Probably both.” (I)*

#### Handling Transitions Through Information

Despite feelings of anxiety when their child was transferred from the pediatric unit, parents felt capable of handling the situation if they were properly informed prior to the transition. Being involved in transfer planning and having an opportunity to visit the new unit or meet the professional team there before the transfer were perceived as positive by the parents.

“*…of course we wanted to stay there and were a bit scared about changing to the ward – but both doctors and nurses helped us to handle our anxiety!” (D)*

Transitions were challenging for the families. Most challenging was the shift from high technological care to an ordinary pediatric unit. One parent stated:

“*Overwhelming, like going from the Hilton to a youth hostel.” (H)*

Parents had a negative experience of the transfer to another unit when they were not informed in advance. They felt overtaken by the decision and, in some cases, did not believe that their child was well-enough to be transferred.

### Participation

#### Establishing a Partnership Based on Sensitivity to Parental Strengths and Vulnerability

Parents described developing a valuable relationship with the professional team and felt included as part of the team. They experienced collaboration with the professional team as efficient, and that the team members were responsive to their wishes when planning the child's care. When parents were exhausted, they felt the members of the professional team were sensitive to the fact that they lacked the energy to participate in the child's care. When this happened, the team cooperated with the parents and offered them space and time for recovery, as well as support from a social worker or psychologist. This helped the family to handle the situation.

“*…We could be involved as much as we wanted without feeling any kind of pressure …” (K)*

However, some parents did not wish to participate in their child's care planning and decision-making because they believed they lacked the required skills.

“*I don*'*t expect to ‘be actively involved in the decision process' when my child is undergoing intensive care.” (O)*

Parents also described occasions when they were not fully satisfied with their child's care, especially when they had wishes that differed from the plans of the professional team. When this occurred, parents felt overlooked. In some cases, they considered that the child received poorer care because their participation was neglected, causing them to feel stressed and frustrated.

“*The staff should listen more to the parents” (Y)*

#### Feeling Valuable When Participating but Needing Encouragement

The parents appreciated being encouraged to participate in their child's care. Being able to do something valuable for their child made them feel important. They stated that the professional team respected the extent to which they wanted to participate, which mainly involved practical tasks such as bathing, feeding, and changing diapers. If they felt confident and ready for more advanced daily care, the professional team supported them step by step, in accordance with the parents' wishes and capabilities.

“*The staff members were very professional and responsive and good at explaining things to us in easy steps so that we felt safe and confident.” (C)*

At the same time parents described that their participation in their child's care depended on the willingness of individual members of the professional team to invite them. Some were more likely to allow them to participate, while others excluded them.

“*A negative aspect when we came here was that I wasn't allowed to be in the room but had to wait in the relatives' room. Which for me was a disappointment not to be there with them and be close.” (F)*

When the staff performed care procedures based on routines and did not include them, they felt excluded as a parent. This was also the case when they were left out of discussions concerning their child's care and treatment.

### Collaboration

#### Unmet Needs Resulting in Suggestions for Possible Improvements

During their child's hospitalization, the parents expressed needs indicating the necessity for improvements in the unit. Parents described a need for a private space where they could be alone to e.g., breastfeed and talk with their spouse as well as a room for resting. The parents would have appreciated been shown around the unit and receiving information about where they could prepare their food, eat or make a cup of coffee. They found it frustrating that they were unable to figure this out until several days after their child had been admitted to the PICU. They expressed that knowing about the existence of these facilities at the ward could have helped them to remain close to their child when they wanted a cup of coffee or something to eat.

The parents wished for more comfortable pillows, duvets, and ergonomic chairs, as they often spent many hours at the PICU. This became even more evident for mothers who had recently given birth.

“*…to the parents, an ergonomic chair or cushion. You are sitting there with your child for hours and want to be close.” (E)*

## Discussion

In the present study, parents' views of FCC were described as “*an incomplete partnership.”* Our findings identified “*partnership*” between parents and HCPs as more evident in some of the framework concepts than in others. According to the Institute for patient and family-centered care, partnership between patients, families, and HCPs constitutes the essence when the core concepts of FCC are achieved (12). As in other care settings, achieving partnership in pediatric intensive care requires an established relationship and collaboration between the child, her/his family, and the HCPs that is grounded in mutuality and where they jointly set and achieve the goals for the child's care (9, 12). In the present study, it appeared that the parents experienced that partnership was partially achieved when they were treated with empathy and respect, and support was provided for the child, parents, and the family as a whole. This is in line with the results from earlier studies, elucidating how emotional support from the professional team strengthens the relationship and interaction between the team and the parents. This form of caring is significant for FCC and considered one of its key aspects (26–28).

Although the parents were generally positive and satisfied with the FCC, a number of areas emerged with potential for development. One such area was the core concept “*Information sharing,”* where there were occasions when parents felt that the professional team withheld information to “*protect”* them or they were given information at very short notice, e.g., before transfer to another unit, which caused them stress and anxiety. Transfer from the PICU to another pediatric unit with a lower level of care is often associated with distress for the parents (29) and they therefore need to be prepared well in advance. In their study, Hakio et al. (30) showed that parents who had been informed and prepared for a transfer in good time had fewer negative experiences. Nevertheless, our findings illustrate that parents were mainly satisfied with the information they received. They found that the professional team adjusted information based on who they were talking to and that siblings were given age-appropriate information. Being well-informed created a sense of security among the parents. This is in line with earlier studies showing that good communication between parents and the healthcare team reduces parental stress and increases satisfaction. It also provides opportunities for parents to participate in decision-making regarding their child's care and treatment. Ultimately, good communication enables both the child and the family to be partners in care based on the principles of FCC (31, 32). Communication is of importance when sharing information. Information provided in the form of one-way communication may not meet the parents' needs. Foster, Whitehead, and Maybee (33) refer to communication as a primary tool for achieving FCC. Working according to FCC principles requires extensive knowledge of person-centered communication, which is a two-way communication style that takes the parents' perspective into consideration when adjusting complete and accurate information to be shared between the parents and HCPs (17, 18).

In the present study, the parents described occasions when the professional team did not collaborate with them about their child's care and treatment. One reason for this may be that due to a high workload the professional team often worked according to routines rather than relation-based care incorporating the parents and child. Shields et al. (34) described various caregiving models, e.g., the professional-led model and the professional-centered model. When these models are employed, HCPs provide care based on the diagnosis and the department's guidelines for the organization of care rather than tailored to the individual needs of the child and her/his family. Support from the organization (35, 36) as well as education about FCC comprising the core concepts and the overall goal of the care model (27, 37) are required for the successful implementation of FCC.

The development of a partnership is dependent on close collaboration between the parents and the HCPs (12). It was evident that when lack of continuity in staffing occurred, the parents in our study became distressed and the relationship established between them and the professionals was interrupted. This finding is consistent with the study by Gill et al. (27). There can be several reasons why continuity is not achieved, for example there may be schematic reasons or staff shortages on the unit. When staff continuity cannot be achieved, this can be bridged by clear individual care plans developed in collaboration with the parents according to the principles of FCC. Individual care plans are often used in palliative care as well as during end-of-life care at the PICU in order to fulfill the parents' and the child's wishes (38).

A strength of the present study is that due to the inclusion criteria there is a variation in the children's length of stay at the PICU. We may have captured a broader view of FCC as the length of stay varied from 2 to 84 days, in addition to the variation in diagnosis. Parents might experience and view FCC differently depending on the severity of their child's condition and whether the care was due to planned surgery, or an acute incident. Another strength is the clear methodological description providing the reader with a possibility to assess the confirmability (39). Regarding pre-understanding, the three authors have no PICU experience, although their background is high technological care ranging from intensive neonatal and adult care to anesthesiology and ambulance care. However, there are some limitations. Parents whose child died during hospitalization were excluded and it is possible that they might have different experiences to those of the parents in the present study. The data were based on five open questions from the EMPATHIC-30 questionnaire. This implies that we were unable to pose follow-up questions or ask for clarification. On the other hand, the parents wrote many detailed descriptions of their experiences, providing rich data for the analysis and thereby the structures of the parents' experiences were extensive. Furthermore, using the information the parents provided through written descriptions allowed us to gain an insight into their experiences without having to request their time for an interview. When a child has been cared for at a PICU, parents are often in a vulnerable situation (4) and an interview might only increase their burden. Data collection was ongoing for a long period, which might have involved changes in care procedures, working methods, and greater awareness of the FCC concept on the part of staff. When discussing transferability it should be borne in mind that PICUs in Sweden and internationally are not designed in the same way, they have different resources, internal policies, and distribution of staff. However, the findings can guide the further development of FCC in pediatric intensive care.

## Conclusion

Overall, the parents described positive experiences of FCC at the PICU. Although parents in general were satisfied with the care during their child's hospitalization, areas for improvement in terms of achieving partnership were identified, mainly in aspects such as participation in decision-making about care and treatment as well as person-centered communication. The input from the parents in our study is valuable and can contribute to the design of interventions for quality improvement in FCC at PICUS. These findings emphasize the importance of using an instrument that focuses on satisfaction with care as the results can serve as a quality indicator of care delivery.

## Data Availability Statement

The datasets presented in this article are not available because the data in the present study is based on sensitive information from parents' when their critical ill child was hospitalized in a pediatric intensive care unit. Although all data is anonymized, it contains information and details that could enable identification of single individuals. According to the ethical approval and the General Data Protection Rules (GDPR) data would only be used for the stated purpose and only be available for the researchers. Therefore, we stated in the in written information to the participants that data will not be shared. Requests to access the datasets should be directed to Karina Terp, karina.terp@med.lu.se.

## Ethics Statement

The studies involving human participants were reviewed and approved by regional ethical review board in Lund (Ref 2018/547, 2019-04602) and was conducted in accordance with the Helsinki Declaration of 2013 (25). The participants provided their written informed consent to participate in this study.

## Author Contributions

This study was designed by PL, KT, and JW. PL led the research as a senior researcher. KT performed the data collection and was responsible for the analysis discussing the result with PL and JW throughout the process and wrote the manuscript. PL and JW contributed with their expertise during the process of writing all parts of the article. All authors read and approved the final manuscript.

## Conflict of Interest

The authors declare that the research was conducted in the absence of any commercial or financial relationships that could be construed as a potential conflict of interest.

## Publisher's Note

All claims expressed in this article are solely those of the authors and do not necessarily represent those of their affiliated organizations, or those of the publisher, the editors and the reviewers. Any product that may be evaluated in this article, or claim that may be made by its manufacturer, is not guaranteed or endorsed by the publisher.

## Abbreviations

PICU: Pediatric intensive care unit
FCC: family-centered care
PFCC: Patient- and Family-Centered Care
HCPs: Health care professionals
EMPATHIC-30: EMpowerment of PArents in THe Intensive Care.
